# Biodegradability and Efficacy of Porous Polycaprolactone Microsphere Dermal Filler for Fine Lines

**DOI:** 10.1111/jocd.70156

**Published:** 2025-04-11

**Authors:** Jin‐Su Kim, Ji‐Hyun Sung, Doo‐Yeon Kwon, Jeong‐Eun Park, Helen Cho, Hye‐Sung Yoon

**Affiliations:** ^1^ Samyang Holding Corp Biopharmaceutical R&D Center Seongnam‐si Republic of Korea

**Keywords:** biodegradable polymer, dermal filler, PCL filler, porous microsphere

## Abstract

**Background:**

Among various filler products designed to improve facial wrinkles, those using biodegradable polymer microspheres have gained attention. Recently, a filler composed of porous polycaprolactone (PCL) microspheres was introduced.

**Aim:**

This study aimed to evaluate and understand the mechanism of PCL biodegradability, safety, and efficacy in reducing wrinkles.

**Methods:**

To examine the degradation characteristics in vitro, the filler product was incubated in phosphate‐buffered saline (pH 7.4) at 37°C, 45°C, and 55°C. Samples from 1 to 104 weeks were obtained to determine changes in the morphology and molecular weight of the porous PCL microspheres. In addition, the product was administered to rabbits to evaluate its in vivo degradability. Treated tissues were sampled at 6, 12, 18, 24, and 30 months to examine the biodegradability of the microspheres. Tissue safety and collagen fiber production were evaluated at the same time points. The anti‐wrinkle effect was evaluated using PRIMOS to measure changes in skin surface roughness in a photoaging mouse model.

**Results:**

In vitro testing revealed that the porous PCL microspheres degraded progressively over time, forming cracks on the surface and showing a decrease in molecular weight. In vivo studies demonstrated that the product degraded safely in tissues and induced collagen formation. Furthermore, skin roughness evaluation using a photoaging mouse model confirmed its anti‐wrinkle effects.

**Conclusion:**

The filler product based on porous PCL microspheres was found to be safely biodegraded in vivo and effectively improved wrinkles.

## 1 Introduction

With the aging population, many individuals seek a healthy and aesthetically pleasing life. Accordingly, various skin beauty products have been introduced to the market, including dermal fillers. Dermal fillers are products injected into wrinkled areas of the skin to improve wrinkles by either physically restoring skin volume or inducing collagen production [1, 2].

Among the various filler products, those based on hyaluronic acid as the main ingredient are the most common. Hyaluronic acid fillers improve wrinkles through physical volume restoration by injecting hyaluronic acid—a natural component of the dermis—directly into the wrinkled areas of the skin. However, they are short‐lived, so BDDE (1,4‐butanediol diglycidyl ether) is used as a cross‐linking agent to prolong their effects. As a result, there is a growing demand for safer and longer‐lasting filler products [3, 4, 5, 6].

Recently, filler products that utilize biodegradable polymers, such as polycaprolactone (PCL) and polylactic acid, have gained significant attention. These fillers do not use cross‐linking agents and last longer than hyaluronic acid fillers, with a duration of effect of 1–2 years. In addition, these fillers are expected not only to provide physical restoration but also to induce autologous collagen production when injected into the skin area of the body [7, 8, 9].

In a previous study, we demonstrated the efficacy and safety of a new filler that uses PCL in the form of porous microspheres as the main component [10]. Through an in vivo study conducted on hairless mice, we observed excellent volume retention for up to 6 months, significant collagen production surrounding the porous PCL microspheres, and no adverse effects on tissue safety during the study period.

As a follow‐up to the previous study, the current study aims to investigate tissue safety following rabbit injection for up to 30 months and biodegradability of the porous PCL microsphere filler product and to examine its anti‐wrinkle effect through in vivo tests on a photoaging hairless mouse model.

## 2 Materials and Methods

### 2.1 Materials

This study used DMFI300 (Lafullen, Samyang Holdings Corp., Korea), a porous PCL microsphere filler product. DMFI300 is a product consisting of a homogeneous mixture of 30%(w/w) porous PCL microspheres and 70%(w/w) sodium carboxymethyl cellulose (Na‐CMC) based gel carrier filled in a 1 cc syringe. The porous PCL microspheres used in the product have a uniform size of 25–50 m and are characterized by multiple pores on the surface and inside of the microspheres. PCL‐EL (EllanseM, Sinclair Pharmaceutical), a commercially available non‐porous PCL microsphere filler, was used as a test control. This product, unlike DMFI300, uses non‐porous PCL microspheres as the main component.

### 2.2 In Vitro Degradation Test

#### 2.2.1 Preparation of Samples

Phosphate buffer saline (PBS) with pH 7.4 and 0.1 N NaOH solution was used for the in vitro degradation test.

To analyze the hydrolysis characteristics of the porous PCL microspheres used in DMFI300, 25 mL of PBS was added to a 50 mL conical tube, followed by the addition of 1 mL of DMFI300. The in vitro degradation test was then performed by shaking at 60 rpm in a water bath at 37°C, 45°C, and 55°C. Samples were collected according to the set intervals (1, 2, 4, 13, 24, 52, 78, and 104 weeks). The collected samples were washed with 25 mL of distilled water to remove impurities. Finally, the collected PCL was subjected to a drying process to remove moisture and used as the sample for analysis.

To investigate the morphological changes of porous PCL microspheres in a harsh environment, 25 mL of alkaline solution (0.1 N NaOH solution) was added to a 50 mL conical tube, followed by adding 1 mL of DMFI300 and shaking at 60 rpm at 37°C [11, 12, 13, 14, 15, 16, 17]. Subsequently, sampling was performed at set intervals (4, 7, 10, 14, and 28 days), washed with 25 mL of distilled water, dried, and then used as samples for analysis.

#### 2.2.2 Analysis of Microsphere Morphology (Scanning Electron Microscopy, SEM)

To determine the morphological changes of the porous PCL microspheres, a field emission‐scanning electron microscope (JSM‐7610F, JEOL, Japan) was used for analysis. Since the sample surface may be damaged during the analysis process, the surface of all samples was coated with platinum for about 80 s using a platinum coater to prevent static electricity. The sample observation was performed at an acceleration voltage of 5 keV, an emission current of 60 A, and a probe current of 6 mA.

#### 2.2.3 Analysis of Molecular Weight (Gel Permeation Chromatography, GPC)

To analyze the changes in the molecular weight characteristics of the PCL used in DMFI300, samples from each condition were analyzed for weight‐average molecular weight and number‐average molecular weight using GPC (PL‐GPC, Polymer Laboratories, USA). Polystyrene was used as the standard material.

The half‐life of PCL molecular weight was calculated using the following equation [18],

$$\ln\;M_{w}=\ln\;M_{w,0}–t/\theta$$

where M_w_ and Mw,0 represent the weight‐average molecular weight at degradation time t and at t = 0 days, respectively, and θ is a constant. The molecular weight half‐life is defined as the time at which M_w_ reaches M_w,0_/2.

#### 2.2.4 Analysis of Chemical Structure (Nuclear Magnetic Resonance, NMR)

To analyze changes in chemical structure due to hydrolysis of PCL used in DMFI300, a 500 MHz NMR spectrometer (AVANCE III HD 500, Bruker, USA) was used. CDCl_3_ and tetramethylsilane were used as the solvent and standard for calibrating chemical shifts, and all experiments were performed at room temperature.

### 2.3 In Vivo Degradation Test and Efficacy Test

#### 2.3.1 Animal Handling

##### 2.3.1.1 Biodegradation Test Using Rabbits

All animal handling procedures were conducted in accordance with the guidelines set by the Institutional Animal Care and Use Committee (IACUC) of the College of Medicine, Chung‐Ang University, ensuring compliance with animal welfare regulations and aiming to minimize animal suffering (No. 201800015, No. 201900049).

Nine‐week‐old New Zealand White rabbits were obtained from Saeron Bio (Gyeonggi‐do, South Korea), and all rabbits were housed in a controlled laboratory environment under optimal conditions: 24°C ± 2°C, 50% ± 10% humidity, and a 12‐h light/dark cycle. Feed was provided ad libitum, and experiments were started after a minimum one‐week acclimatization period.

##### 2.3.1.2 Hairless Mouse UV Photoaging Model

All animal experiments were conducted after obtaining approval from the IACUC of the College of Medicine, Chung‐Ang University (No. 202000098). Six‐week‐old SKH1‐Hrhr hairless mice were obtained from Orient Bio (Gyeonggi‐do, South Korea), and all mice were housed in a controlled laboratory environment under optimal conditions: 24°C ± 2°C, 50% ± 10% humidity, and a 12‐h light/dark cycle. Feed was provided ad libitum, and experiments were started after a minimum one‐week acclimatization period.

#### 2.3.2 Test Protocol

##### 2.3.2.1 Biodegradation Test Using Rabbits

A total of 10 rabbits were anesthetized by intramuscular injection of a mixture of zoletil (30 mg/kg) and rompun (10 mg/kg). Subsequently, 200 μL of DMFI300 was injected subcutaneously into three sites on the dorsal region. Afterward, two rabbits were euthanized at each time point (6, 12, 18, 24, and 30 months), and tissues from the injection sites were harvested to assess tissue safety, collagen production, and biodegradation of the administered samples.

##### 2.3.2.2 Hairless Mouse Ultraviolet Photoaging Model

To induce photoaging‐related fine wrinkles in hairless mice, ultraviolet B (UVB) radiation was applied using BIO‐SPECTRA (Viber Rourmat, France). UVB was applied at 30–100 mJ/cm^2^ two to three times per week for 24 weeks, with a total dose of 2040 mJ/cm^2^ during the study.

Both UVB‐untreated and 24‐week UVB‐irradiated animals were anesthetized with a mixture of zoletil and rompun (3:1, v:v). Subsequently, photoaging mice received a single subcutaneous injection of 100 μL of PBS, DMFI300, or PCL‐EL in the lower midline region of the dorsal area (Table 1). Wrinkle changes before and after filler injection were analyzed using PRIMOS^LITE^ (GHM Messtechnik GmbH, Germany) at eight time points (immediately after filler injection, 1 day, 3 days, 1 week, 2 weeks, 4 weeks, 8 weeks, and 12 weeks) (*n* = 5).

**TABLE 1:** Photoaging model group.

	UV irradiation	Injection substances
Group 1	Non‐irradiation	—
Group 2	Irradiation	Control, PBS
Group 3		DMFI300
Group 4		PCL‐EL

#### 2.3.3 Histological Evaluation

##### 2.3.3.1 Biodegradation Test

After euthanizing the experimental animals, skin tissue from the filler injection site and normal tissue without injection were harvested and fixed in a 10% neutral buffered formalin solution. Subsequently, 5 μm sections were made and examined using hematoxylin and eosin (H&E) staining to assess the presence of residue.

##### 2.3.3.2 Safety Test

After euthanizing the experimental animals, skin tissues from the filler injection site were harvested and fixed in a 10% neutral buffered formalin solution. Subsequently, 5 μm sections were made, and H&E staining was performed to assess inflammation and foreign body reactions. Light microscopy was used to evaluate the degree of inflammation and foreign body reaction, which was graded on a four‐point scale [19].

##### 2.3.3.3 Effectiveness Test

Skin tissue from the filler injection site was harvested, fixed in 10% neutral‐buffered formalin solution, and sectioned at 5 μm. Masson's trichrome staining was used to assess the degree of collagen fiber production, and immunohistochemistry (IHC) staining was performed to identify collagen types I and III.

#### 2.3.4 Wrinkle Analysis

To analyze the changes in wrinkles over time after filler injection, PRIMOS 5.8 software was used. Among the roughness parameters that can be used to assess wrinkle changes, *R*_t_ (m, the vertical distance between the highest and lowest points of the generated wrinkles) and *R*_max_ (m, the largest of the consecutive values of *R*_t_ calculated over the evaluation length) were measured.

## 3 Results

### 3.1 In Vitro Degradation

#### 3.1.1 Changes in the Morphology of Porous PCL Microspheres

**FIGURE 1:**
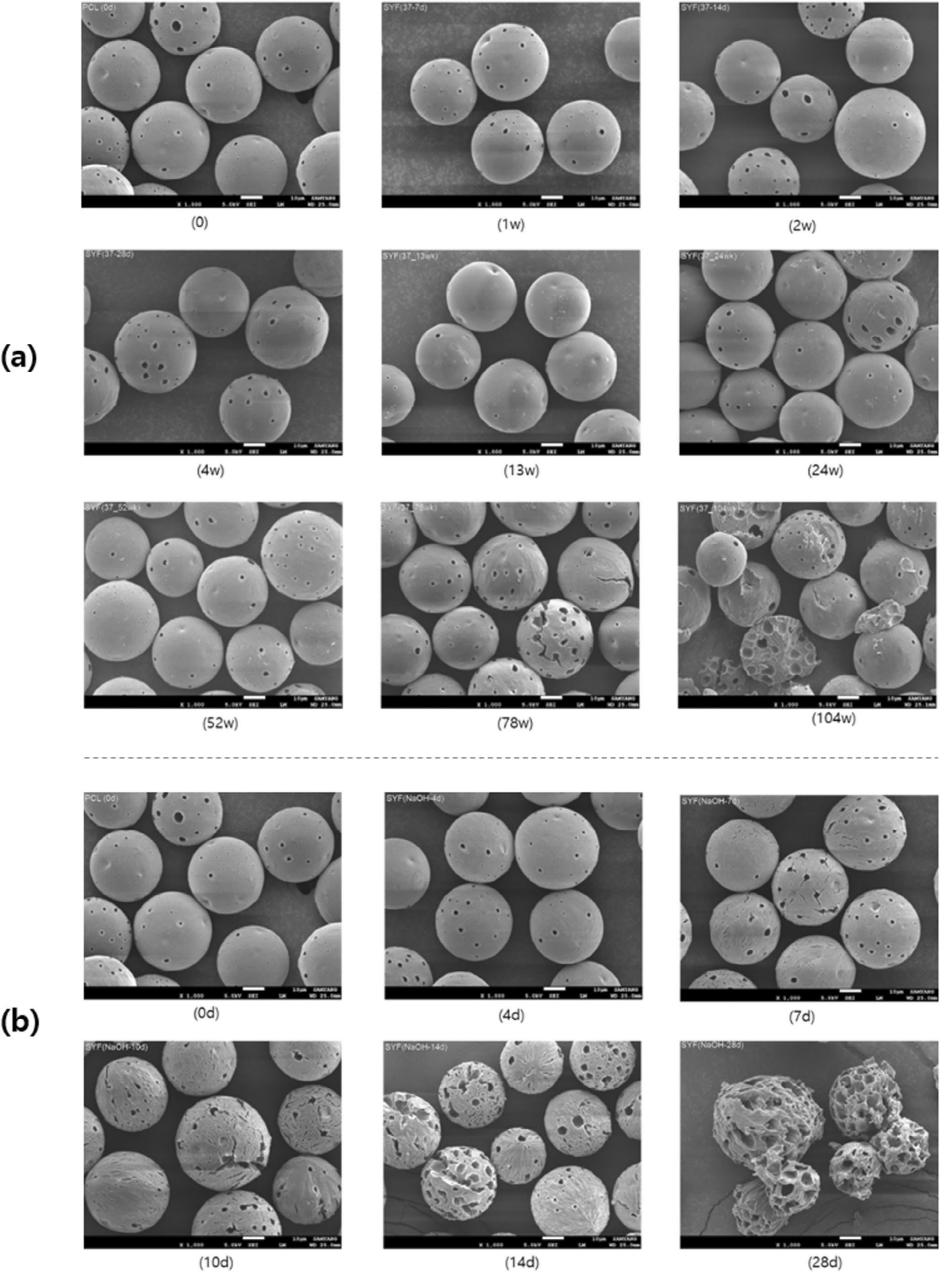
SEM Images of PCL microsphere in (a) PBS and (b) 0.1 N NaOH solution at 37°C.

To check the morphological degradation patterns of porous PCL microspheres, in vitro degradation tests were conducted, and samples collected at different time points were analyzed using SEM. First, the morphology of the samples collected under PBS storage conditions at 37°C showed no significant changes in the morphology of the porous PCL microspheres until week 52. However, cracks began to appear on the surface of the microspheres from week 78, with complete cracking observed by week 104 (Figure 1a).

Additionally, we aimed to induce rapid degradation of PCL microspheres through accelerated testing in order to observe the morphological degradation patterns. In the SEM images of the samples collected in 0.1 N NaOH solution, the microspheres in the harsh condition developed microcracks on the surface starting from day 7, which expanded over time (Figure 1b). By day 28, multiple cracks had formed, and the pores appeared enlarged compared to their initial state.

#### 3.1.2 Changes in Molecular Weight and Chemical Structure

**FIGURE 2:**
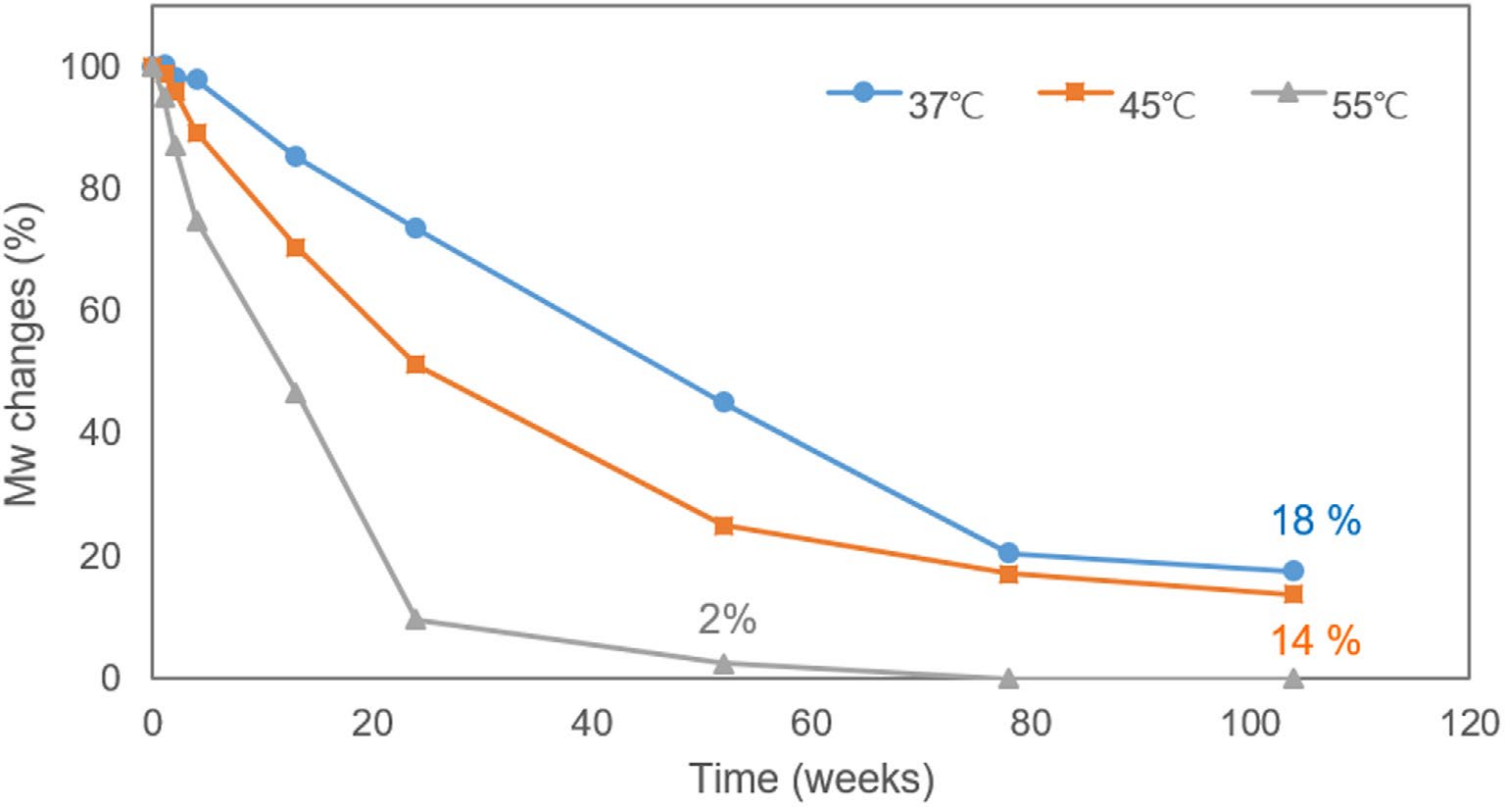
Weight‐average molecular weight changes of PCL microspheres at 37°C, 45°C, and 55°C.

The changes in molecular weight of PCL over time were measured using GPC. To identify the time point of complete degradation of porous PCL microspheres, the changes in molecular weight were plotted under 37°C, 45°C, and 55°C conditions (Figure 2). The results indicated that the weight‐average molecular weight (M_w_) half‐life of the porous PCL microspheres was approximately 52 weeks at 37°C, 24 weeks at 45°C, and 13 weeks at 55°C. At week 104, the molecular weight had reduced to 18% of the initial value at 37°C and 14% at 45°C, while it fell to less than 3% by week 52 at 55°C.

**FIGURE 3:**
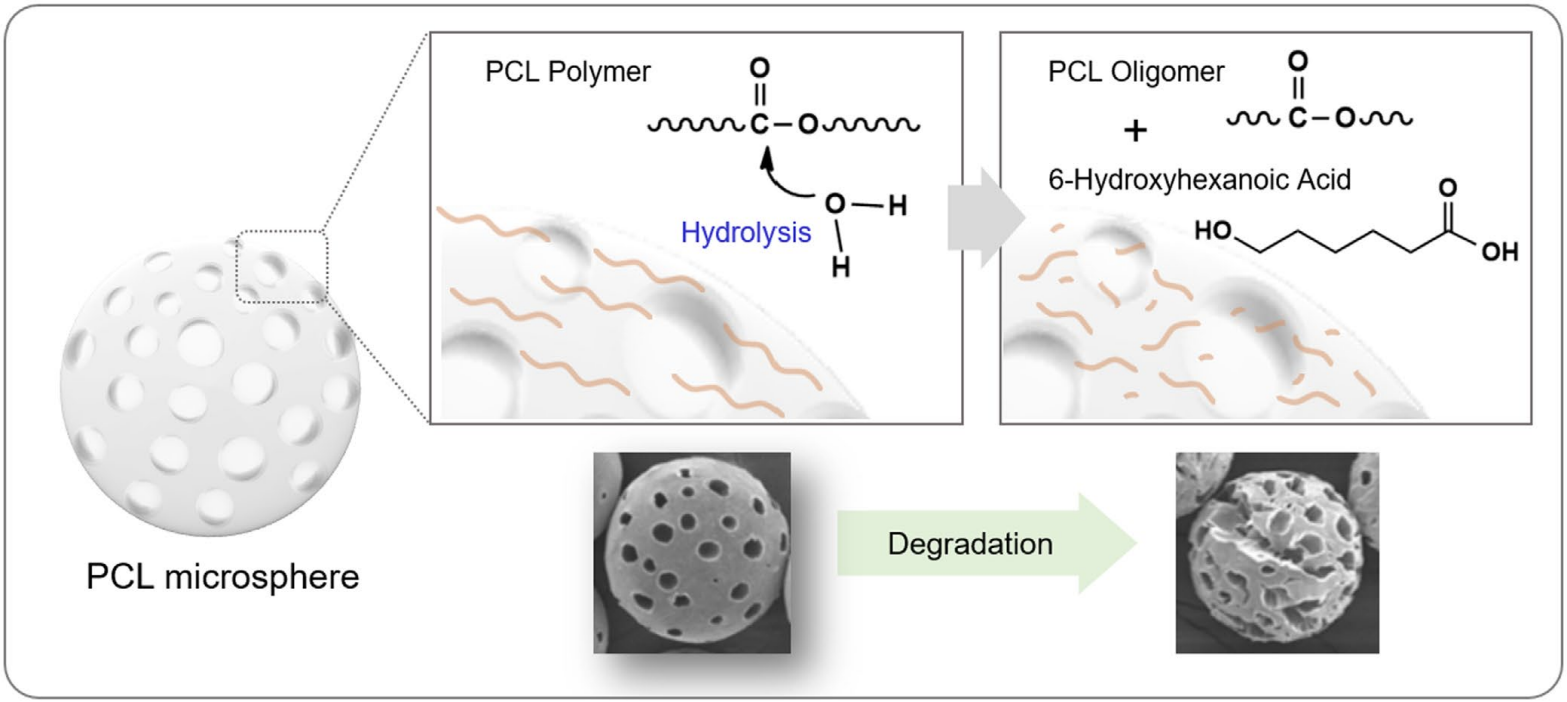
Schematic showing the degradation mechanism of PCL microsphere.

We further investigated the chemical structural changes of the polymer resulting from the degradation of PCL microspheres. During hydrolysis, the PCL polymer undergoes chain scission, leading to the formation of oligomers. A schematic diagram illustrating the chemical chain scission of the PCL polymer is presented (Figure 3).

**FIGURE 4:**
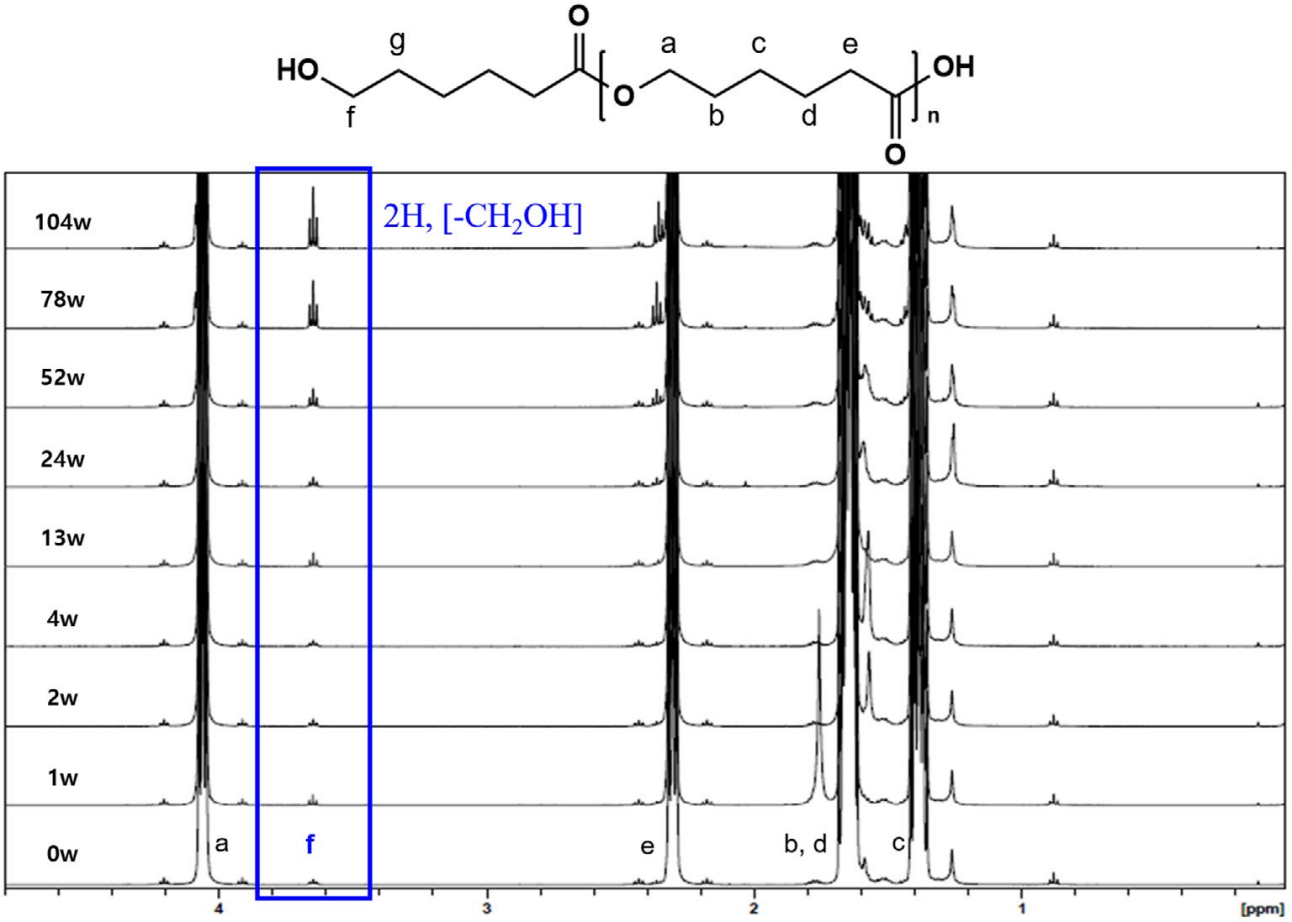
^1^H‐NMR spectra of PCL microsphere at 37°C in PBS with in vitro time.

The confirmation of polymer chain scission and its associated structural changes was carried out through NMR analysis (Figure 4). The protons of the repeating unit —CH_2_— in the PCL structure are observed as broad peaks at positions a, b, c, d, and e. Meanwhile, the proton peak of the —CH_2_OH group located at the terminal position (f) is shifted to 3.65 ppm due to the influence of the —OH group. This peak appears as a triplet in accordance with the *n* + 1 rule, where n represents the number of hydrogens on the adjacent carbon atom (g).

As the degradation of the PCL polymer progresses, chain scission increases the number of molecules. This was confirmed by observing changes in the peak corresponding to the polymer's terminal group (—CH_2_OH) in the NMR spectrum. Specifically, the peak around 3.65 ppm (triplet, highlighted in the blue box) corresponds to the two protons of the —CH_2_OH group at the terminal of the PCL polymer. The triplet splitting pattern arises due to spin–spin coupling with adjacent methylene (—CH_2_—) protons, indicating that the terminal hydroxyl (—OH) group remains chemically stable throughout the degradation process. The increasing intensity of this peak over time confirms the progressive cleavage of polymer chains, as more terminal —CH_2_OH groups are exposed without altering the local chemical environment.

Furthermore, no significant change in the peak area was observed up to the 52‐week sample; however, from the 78‐week sample onward, a sharp increase in the peak area was observed, confirming a degradation pattern that aligns with the molecular weight distribution results obtained from GPC.

### 3.2 In Vivo Degradation Test

#### 3.2.1 In Vivo Biodegradability

**FIGURE 5:**
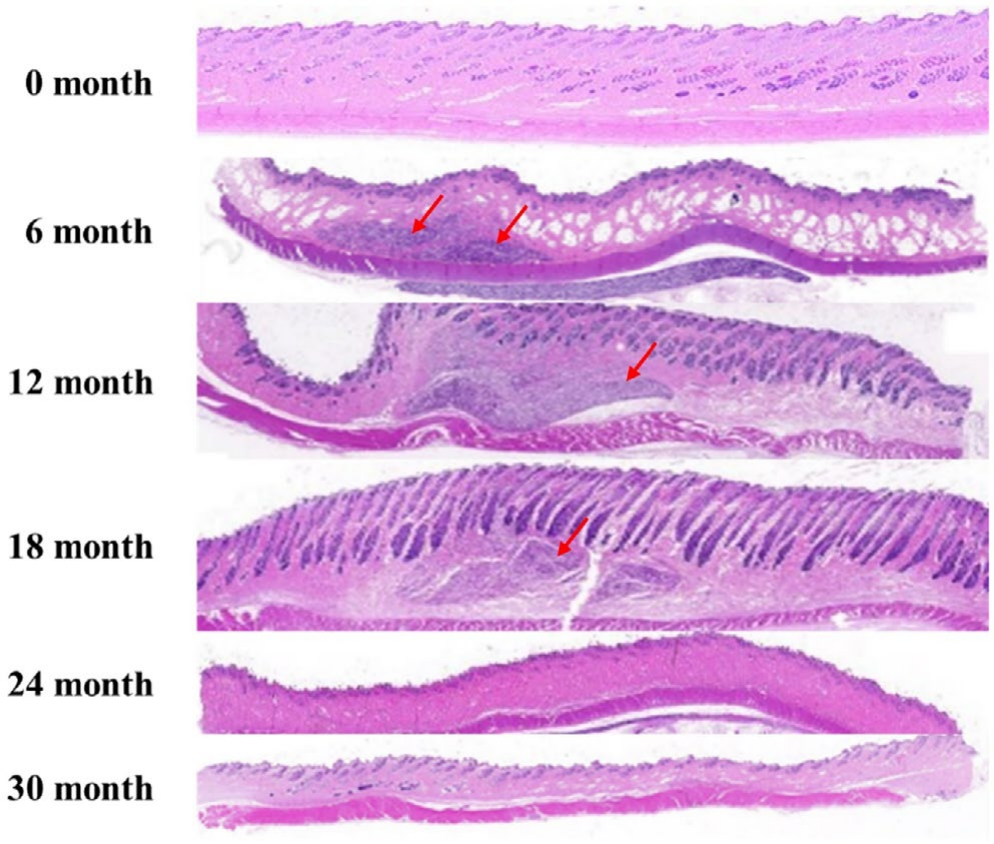
Confirmation of residual substances over time after DMFI300 injection—H&E stain (×7). Red arrow: DMFI300.

To assess the safe degradation of the product in vivo, skin tissue from the filler injection site was harvested at each time point following the injection of DMFI300 into rabbit skin, and the presence of residues was evaluated. Examination of tissue slides at 100× magnification using light microscopy revealed the presence of residual polymer microspheres in DMFI300 up to 18 months. By 24 months, most microspheres had biodegraded, and all injected in vivo samples were biodegraded by 30 months (Figure 5).

#### 3.2.2 Evaluation of Safety in Tissue

**FIGURE 6:**
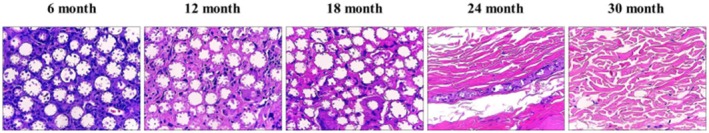
Evaluation of foreign body and inflammatory responses over time after DMFI300 injection—H&E stain (×400).

Filler‐injected tissues were harvested and evaluated for tissue safety after H&E staining. No significant inflammatory reaction was observed at any time point [19]. A mild inflammatory response (score 1), characterized by the presence of a small number of lymphocytes and giant cells in fibrous tissue, was noted following DMFI300 injection (Figure 6).

#### 3.2.3 Detection of Collagen in the Tissue

**FIGURE 7:**
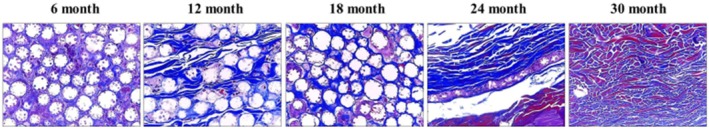
Evaluation of collagen fiber formation over time after DMFI300 injection—MT stain (×400).

**FIGURE 8:**
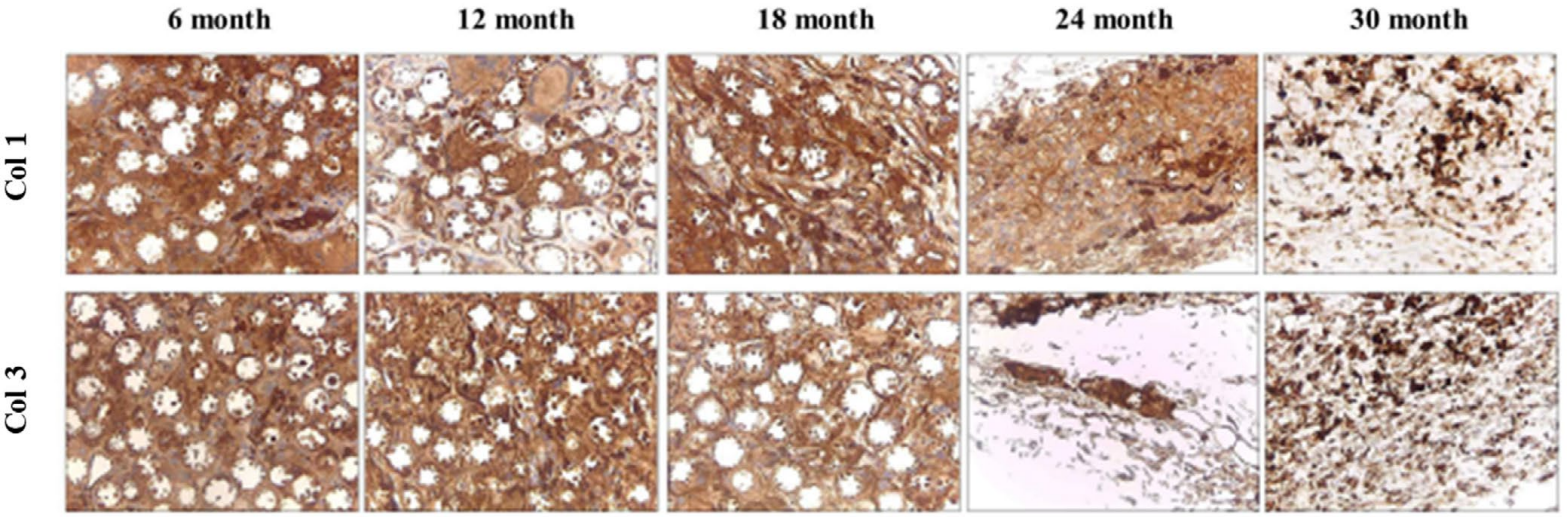
Evaluation of collagen types I and III formations over time after DMFI300 injection—IHC stain (×400).

To examine the formation of collagenous fibers and collagen types I and III in filler‐injected skin tissue, Masson's trichrome (MT) staining and IHC were performed. MT staining showed an increase in collagen fibers between the microspheres from the time of DMFI300 injection until 24 months (Figure 7). Additionally, IHC analysis revealed abundant collagen types I and III around the microspheres, confirming active collagen production (Figure 8).

### 3.3 Wrinkle Analysis in the UV Photoaging Mouse Model

**FIGURE 9:**
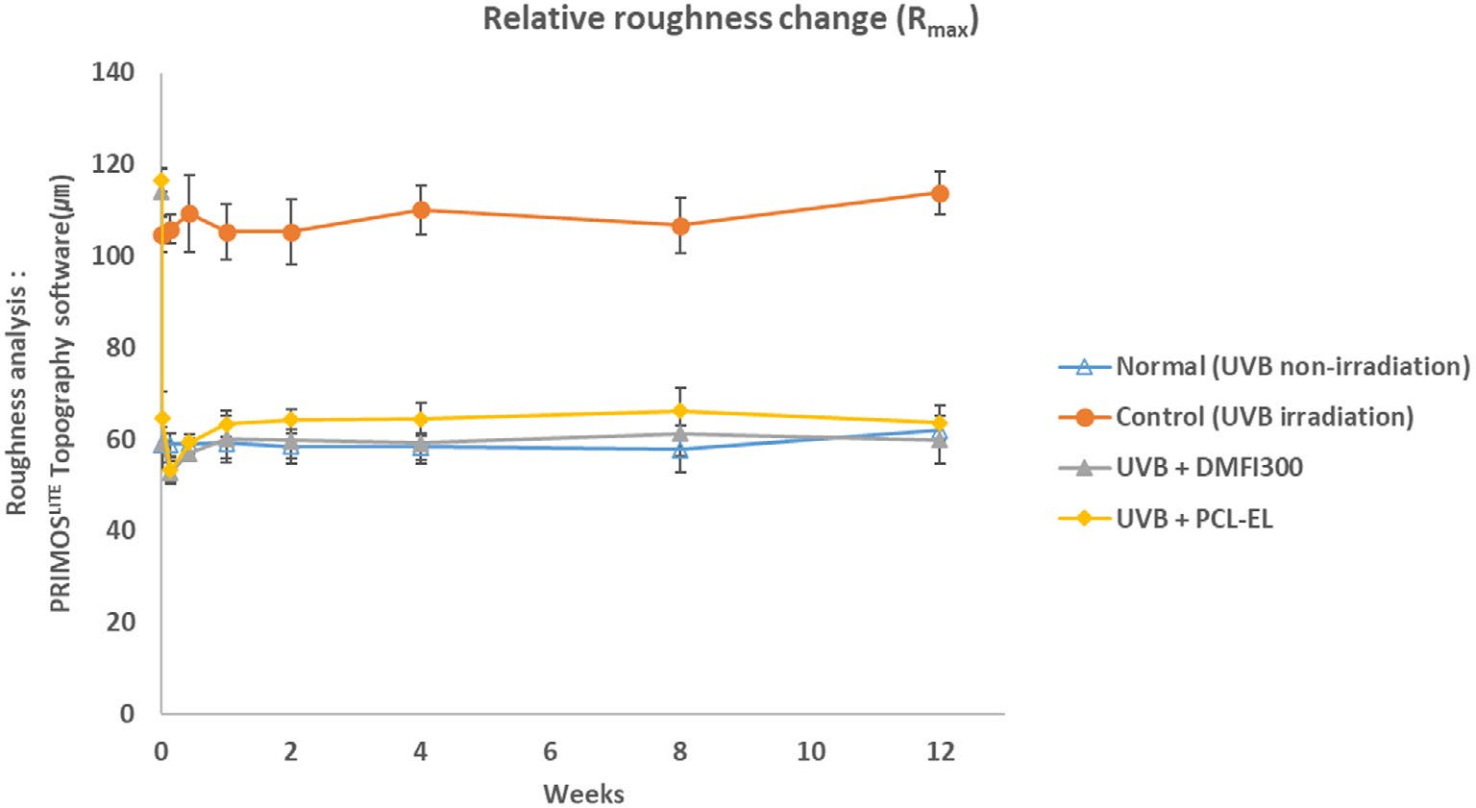
Evaluation of *R*_max_ change before and after filler injection, PRIMOS^LITE^ Topography (with Software PRIMOS 5.8).

**FIGURE 10:**
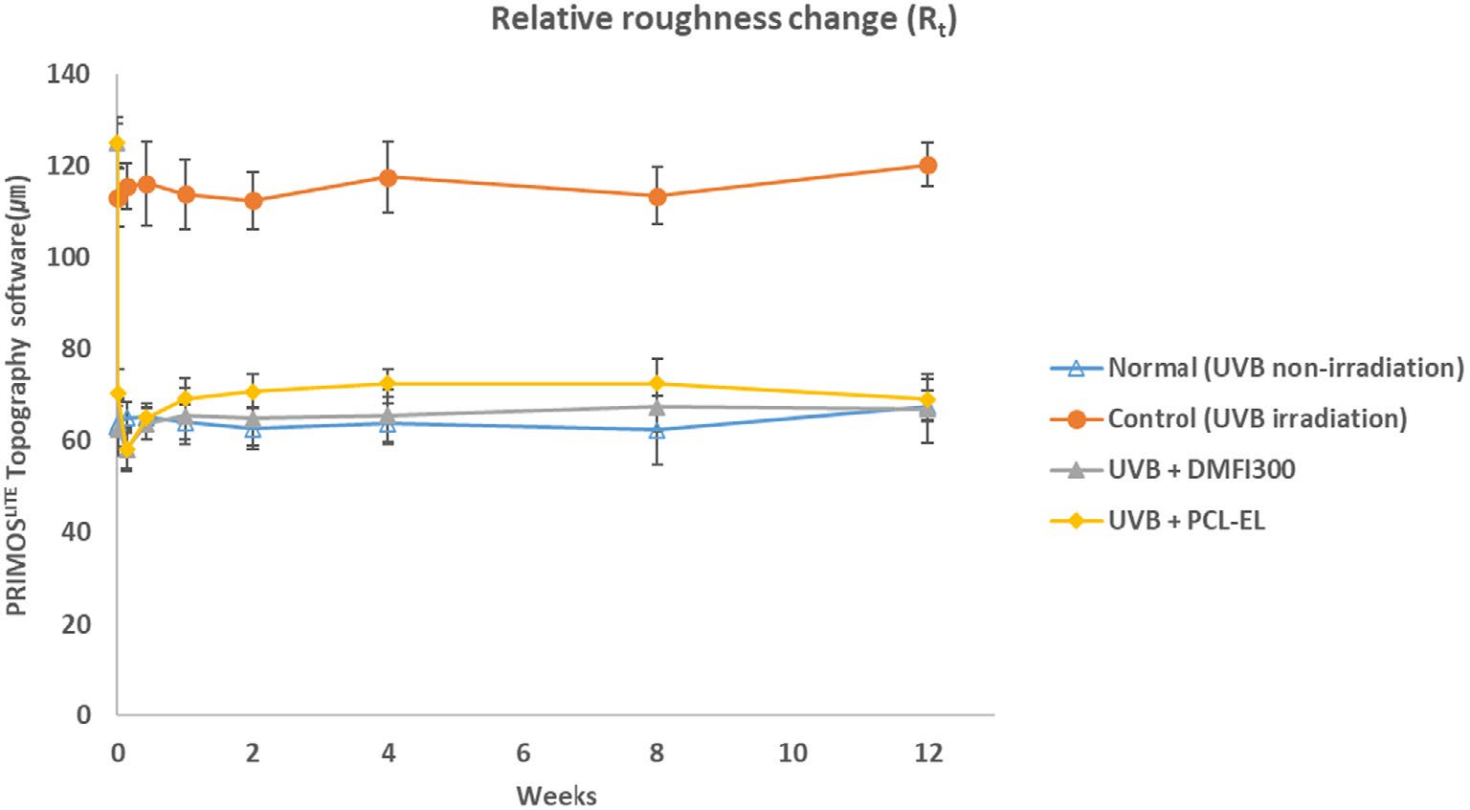
Evaluation of *R*_t_ change before and after filler injection, PRIMOS^LITE^ Topography (with Software PRIMOS 5.8).

In a UV‐induced photoaging mouse model, changes in skin roughness following the injection of DMFI300 and PCL‐EL were compared over time. A 48.32% reduction in *R*_max_ and a 49.95% reduction in *R*_t_ were observed in the DMFI300 group, while a 44.58% reduction in *R*_max_ and a 43.84% reduction in *R*_t_ were observed in the PCL‐EL group. Additionally, when comparing wrinkle changes immediately before and 12 weeks after injection, the DMFI300 group demonstrated a reduction in *R*_max_ of 47.52% and in *R*_t_ of 46.47%, whereas the PCL‐EL group showed reductions in *R*_max_ of 45.48% and in *R*_t_ of 44.75% (Figures 9 and 10).

## 4 Discussion

With the aging population, various products are being introduced to the market to improve skin volume loss and reduce wrinkles. In particular, products using biodegradable polymers that can be safely degraded in the body have attracted great attention recently.

Among the various biodegradable polymers, PCL is a safe material that has been used for various medical devices and is FDA‐approved as a biocompatible polymer. PCL is an aliphatic polyester with a degradation period suitable for use in filler formulations and has the advantage of controlling the degradation period to a certain level by regulating the molecular weight [11].

While biodegradation research has been conducted on various medical products using PCL, few studies have focused on the biodegradability of porous PCL microspheres. Thus, we attempted to characterize the biodegradability in vitro and in vivo of a filler product that uses porous PCL microspheres as the main ingredient. In addition to the volume restoration efficacy after filler injection, which was confirmed in previous studies, we also investigated the anti‐wrinkle effect by evaluating the skin roughness after filler injection.

First, we conducted an in vitro degradation study of porous PCL microspheres to determine the changes in the morphology of the microspheres over time under various temperature conditions. The results showed that the porous PCL microspheres cracked and degraded over time and that this degradation characteristic accelerated as the ambient temperature increased.

At 37°C, which is closest to human body temperature, we found that the morphology of PCL remained stable until week 52 and then changed rapidly from week 78. GPC results also indicated a rapid decline in the molecular weight of PCL during this period.

Next, we tested whether the filler product could be safely degraded in vivo. After injecting the filler product into rabbits, tissue samples were taken from the injection site at regular intervals, and the PCL microspheres were checked for residual PCL in the tissue. The results showed that PCL microspheres were clearly observed by month 18, and more than 90% of the microspheres had disappeared by month 24. At the final time point of 30 months, no microspheres were observed, and no abnormalities were found in the tissue. Moreover, when microspheres were observed, all of the inflammatory responses were mild (score 1). Therefore, this study demonstrated that DMFI300 was mostly safely degraded within 24 months after injection in the body.

Collagen production following DMFI300 administration was observed around the porous PCL microspheres. Extensive literature shows that filler products using biodegradable polymer microspheres induce a mild inflammatory and foreign body reaction, leading to encapsulation with collagen [20, 21, 22].

Finally, we previously confirmed the volume retention efficacy of DMFI300 in naïve hairless mice [10], and its efficacy was also demonstrated in clinical trials (Chung‐Ang University Hospital, Korea, IRB no. 1861‐001‐328). In the next phase, to evaluate the wrinkle improvement effect of DMFI300 in an aged hairless mouse model, we induced photoaging through UVB irradiation. Prolonged sunlight exposure causes photoaging of the skin, leading to dryness, wrinkles, and pigmentation. Among various types of UV radiation, UVB has greater energy than UVA and UVC and is known to cause over 90% of erythema, inflammation, and skin cancer associated with UV exposure [23]. While photoaging animal models have limitations in fully representing the overall aging process, they are useful for studying skin damage induced by UV radiation and for developing treatments. Therefore, the test method of assessing wrinkle changes after a test article injection to UVB‐induced photoaging hairless mice has been widely reported as a representative in vivo model to evaluate the efficacy of dermal fillers [24, 25]. In this study, we confirmed the efficacy of skin roughness improvement following DMFI300 administration using the UV photoaging mouse model.

This study confirmed the tissue safety, biodegradability, and specific mechanisms of wrinkle improvement through tissue regeneration of the porous PCL microparticle filler product, with clinical efficacy [26] and safety in tissue repair demonstrated through in vitro and in vivo experiments. Further studies are needed to evaluate the biodegradability of this product in clinical applications, extending beyond animal models.

## Author Contributions

Jin‐Su Kim and Hye‐Sung Yoon conceptualized and designed the study. Ji‐Hyun Sung, Doo‐Yeon Kwon, and Jeong‐Eun Park were responsible for data collection and analysis. Jin‐Su Kim, Ji‐Hyun Sung, Doo‐Yeon Kwon, and Jeong‐Eun Park drafted the manuscript. Helen Cho and Hye‐Sung Yoon supervised the study and validated the results. All authors reviewed and revised the final manuscript and share responsibility for all aspects of the study.

## Ethics Statement

All animal experiments in this study were conducted with the approval of the Institutional Animal Care and Use Committee (IACUC) of Chung‐Ang University College of Medicine under the following protocols: No. 201800015, No. 201900049, and No. 202000098.

## Conflicts of Interest

The authors declare no conflicts of interest.

## Data Availability

The data that support the findings of this study are available from the corresponding author upon reasonable request.
